# New Biological Morphogenetic Methods for Evolutionary Design of Robot Bodies

**DOI:** 10.3389/fbioe.2020.00621

**Published:** 2020-06-19

**Authors:** Nick Hockings, David Howard

**Affiliations:** Robotics and Autonomous Systems Group, Cyber-Physical Systems, Data61, Commonwealth Scientific and Industrial Research Organization (CSIRO), Pullenvale, QLD, Australia

**Keywords:** morphogenetics, mutation-of-mutability, evolvability, epigenetics, cell-lines, remodeling, morphogens, macro-evolution

## Abstract

We present some currently unused morphogenetic mechanisms from evolutionary biology and guidelines for transfer to evolutionary robotics. (1) DNA patterns providing mutation of mutability, lead to canalization of evolvable bauplans, via kin selection. (2) Morphogenetic mechanisms (i) Epigenetic cell lines provide functional cell types, and identification of cell descent. (ii) Local anatomical coordinates based on diffusion of morphogens, facilitate evolvable genetic parameterizations of complex phenotypes (iii) Remodeling in response to mechanical forces facilitates robust production of well-integrated phenotypes of greater complexity than the genome. An approach is proposed for the tractable application of mutation-of-mutability and morphogenetic mechanisms in evolutionary robotics. The purpose of these methods, is to facilitate production of robot mechanisms of the subtlety, efficiency, and efficacy of the musculoskeletal and dermal systems of animals.

## 1. Introduction

From the perspective of biology and medical sciences it is evident in the robotics literature to date, that there is little awareness in the robotics community of what is known about (i) how bodies work at a material and mechanical level, (ii) how bodies vary vs. what parameters are highly conserved across species, (iii) the mechanisms of biological morphogenetics which produce highly functional, evolvable bodies.

As a step toward remedying the situation, this paper presents four mechanisms that are critical to how biological morphogenetics generates the diversity and sophistication of bodies seen among mobile multicellular animals, and how they might be implemented for robotics. It is hoped that this will encourage roboticists to invest the time necessary to study the relevant areas of biology thoroughly.

The biological terms used in this article are available in general English dictionaries (e.g., Merriam-Webster, 2020a). For those seeking more of the biological background, we have provided a reference document (Hockings and Howard, 2019), which provides a concise path from an undergraduate start point, up to the current orthodox understanding in biological morphogenetics. This paper uses an information theoretic view of evolution. A useful introduction to the relevant information theory concepts is provided by Prokopenko et al. (2009).

### 1.1. Motivation for Biological Realism

It has been claimed within evolutionary robotics that “evolutionary methods provide a successful approach to designing robots" (Jelisavcic et al., 2019). However, so far the technique is little used by those who design robots for purposes other than researching evolutionary robotics. Here we are concerned with defining the materials and geometry of robots, as opposed to combining human designed parts, or creating controllers. If the technique is to become sufficiently effective to be widely used, then substantial improvement in the designs it produces is required.

There are in fact few papers reporting robots where the materials, geometry, and topology of the mechanism of the robot are substantially evolved. Lipson and Pollack (2000), the first demonstration of evolution of robotic hardware, involved modules of rods that could be telescopically actuated and evolved to form various truss-like bodies capable of some locomotion.

Hiller and Lipson (2010) evolved “amorphous” soft robots in simulation. The simulation represented a robot made of multiple materials, some of which could expand and contract. Discrete cosine transforms, compositional pattern producing networks and Gaussian mixtures were trialed as representations for the evolution. Hiller and Lipson (2011) developed a manufacturing process for soft robots consisting of voxels of closed-cell foam that could be actuated by varying the external pressure. This enabled manufacture and actuation of this class of evolved soft robots

Rieffel et al. (2014) evolved a caterpillar robot in simulation, with homogeneous finite element mesh actuated by virtual “muscles.” The shape, stiffness, and damping of the body material, and the muscle placement were evolved. There was no direct transfer of the evolved design to a mobile physical robot. A “face encoding L-system” developmental encoding was used for the tetrahedral mesh of the body shape, producing repeated body segments. Later versions were actuated by varying the stiffness of the finite element mesh. Structure was created by an artifact of mesh resolution, that larger tetrahedra have less scope for flexing, so behave as stiffer than their nominal modulus.

Vujovic et al. (2017) evolved soft printed legs for motor modules. The legs were defined by length, thickness and angle. The topology of the parameterization was not evolved.

The principal group building evolved soft robot bodies has been Cheney and collaborators. Cheney et al. (2014) developed the Voxelize simulator for closed cell foam robots, and Cheney and Lipson (2016) applied Compositional Pattern Producing Networks with Neuro-Evolution of Augmenting Topologies (CPPN-NEAT) for evolution. The use of CPPN-NEAT produced contiguous blocks of voxels of the same material and a more compact parameterization than evolution on voxels directly. Kriegman et al. (2018b) demonstrated modification in response to mechanical forces—with a simulated robot growing “calluses.” Kriegman et al. (2018a) introduced simulations of soft voxel robots that change voxel specifications and consequently overall shape over their “lifetime.” Kriegman et al. (2019) introduced a new manufacturing approach with hollow interconnected silicone voxels, pneumatically actuated via an airline. These robots were no longer confined to a pressure chamber.

#### 1.1.1. Advantages and Disadvantages of Existing Techniques

The methods above are a start, but the robots they produce are extremely limited. Robot mechanisms of the subtlety, efficiency, and efficacy of the musculoskeletal and dermal systems of animals, require something else.

Bringing into the body plan ideas like radial and bilateral symmetry give us forms that are more likely to be useful. Auerbach and Bongard (2012) demonstrated the use of CPPNs can produce radial and bilateral symmetry, and symmetry of repeated parts. However, these CPPNs do not constrain the variation to viable phenotypes. This is a limitation in developing complex bodies in which many parts must mechanically work together. The necessary mutual information between parts is not conserved under mutation by CPPNs. This can result in a high proportion of non-viable phenotypes among offspring.

The papers cited above do not address (i) the class of fibro-elastic structures that characterize the anatomy of highly mobile multicellular animals such as vertebrates, arthropods, and molluscs, and (ii) the constrained variation required to consistently produce viable offspring after mutation. Systems such as CPPN-NEAT are Turing complete, so in principal could represent any computable function. However, the question is whether a particular representation provides an efficient and intuitive way of performing a particular task.

Conversely biological emulation has enabled progress. For example, evolution of bodies exhibiting repetition with variation was demonstrated by Doursat (2009) and Doursat et al. (2012) using the MapDevo3D “Modular Architecture by Programmable Development,” (Doursat and Sánchez, 2014). MapDevo3D provides a particle based 3D soft matter simulator combined with genetic regulatory network (GRN) control of automata behavior of the growing, dividing, actuating particles.

The benefits of GRN control of automata as a means of specifying morphogenesis are discussed further in the context of epigenetics in section 2.2.1.

### 1.2. Reverse Engineering

In robotics there is a commonly asserted rejection of reverse engineering, e.g.,“*We think blind copying is exactly what you don't want to do*.” Full cited in Taubes (2000). This view is at odds with the reputation across engineering as a whole, for the efficacy of reverse engineering, as a means of acquiring understanding of a new technology from a working example (Wang, 2010). The fundamental technique of reverse engineering is to reproduce the parts with sufficient precision that the duplicate system assembled from them, reproduces the function of the original.

It is imperative that anyone wishing to reproduce a function observed in biology, should thoroughly familiarize themselves with what is known in the biological sciences about how that function is implemented in nature. Most importantly when an engineer proposes to reproduce a function, using a dissimilar causal mechanism to the original, then the engineer must explain why the different design should be expected to work.

#### 1.2.1. What This Paper Does and Does Not Advocate

We do not advocate emulating all observed features of biology. Rather we are advocating four specific mechanisms that are responsible for a critical part of the biological morphogenetic system. These are the high level causal understanding of the mechanisms, not the full biochemical complexity as they exist in living species. There is inductive reason to believe that these mechanisms are effective, because they are the causal mechanisms proven to exist in the working example.

### 1.3. Domain Specificity

Evolutionary algorithms can be expressed as search algorithms, i.e. “find examples of higher fitness” by some definition of “fitness” (which may include novelty). Better than random performance by search algorithms is intrinsically domain specific. This is because “*how well any search algorithm performs is determined by how well it is ‘aligned’ with the distribution P(f) that governs the problems on which that algorithm is run*.” (Wolpert, 2012). Such “alignment” implies mutual information between the algorithm and the problem. This mutual information constitutes the prior expectations contained in the algorithm.This applies regardless of whether the priors are explicitly expressed or implied in the structure of the algorithm (Prokopenko et al., 2009).

### 1.4. Parameterization

A critical, but often neglected prior is the choice of how to parameterize a problem. This can readily change the search space from (i) high mutual information between samples (e.g., spaces with smooth gradient and curvature)—allowing algorithms that find very good solutions from a small number of samples, to (ii) white noise, where samples have no mutual information (i.e., a parameterization that has no correlation with the solutions), to (iii) a space that is pathological for a given class of algorithms (e.g., decoupling of parameters that must co-vary in the set of viable solutions—so adding many dimensions to the search space).

A number of non-biological “short-cuts” in artificial evolution have been proposed. For details see Stanley and Miikkulainen (2003). Some of these are not always the advantage they may appear to be, because they decouple important sources of information guiding well balanced development of the phenotype. “*Cartesian coordinates*” for patterning amount to global coordinates, which lack implied information about the relative position and scale of other parts. “*Instantaneous spreading of a canvas of cells*,” loses mechanisms which parameterize the initial structure. “*Real time as a regulator of gene expression*” lacks information about what the affected cells and other interacting cells have done. Often the relative timing is more important in sequential development, as it determines what other information is available, e.g., in the layering of structures.

### 1.5. Applicable Domain of the Proposed Methods

The methods proposed provide a means to specify evolvable parameterizations of mechanical systems composed of interdependent parts. They enable low dimensional descriptions of the variability and conserved mutual information characteristic of the anatomy of animals. This is expected to be most relevant to functional emulation of anatomical structure. In this regard, the subtleties of fibrous soft tissue anatomy are likely to be beneficial to the design of robots (for example the review of hand anatomy and the manufacturing techniques in Hockings, 2016). In particular the functionality of ligamentous joints, anatomical tendon networks, and dermal structures has not been matched in robotics. While hands are arguably the least satisfactory component of current robots, these techniques are applicable to emulation of all parts of the musculoskeletal and dermal systems.

## 2. Translatable Mechanisms—From Biological Morphogenetics

Here the four mechanisms from biological morphogenetics, that we propose would be beneficial to evolving the bodies of robots, are presented.

### 2.1. Mutation of Mutability–for Evolution of Evolvability

DNA is only 1% protein-coding genes (U.S. National Library of Medicine, 2020). The non-coding DNA contains many repeating patterns that affect the probability of crossing over events (the predominant form of mutation). Also in the non-protein-coding DNA are the “cis-regulatory elements" that regulate the activity of the genes (Narlikar and Ovcharenko, 2009). The effect of crossing over events is mostly to add or remove cis-regulatory elements, adjusting whether and how much the transcription rate of a gene responds to a stimulus.

The existence of DNA patterns that affect the likelihood of mutations (McClintock, 1950; King, 2012), and can themselves be added or removed by mutation, enables gene specific mutation of mutability. This has effect through kin-selection. That is if mutations of a given gene have a high probability of non-viable off-spring, then mutations which reduce the mutability of the gene are selected for. Conversely all improvements of genetically induced fitness are dependent on DNA change and therefore elevated mutability of those genes capable of producing improvements in fitness—thus tending to provide a founder effect (Box 1) of high mutability of genes associated with recent improvements in sfitness.

Box 1 **Definitions of biological terms.**

Bauplan: “The generalized structural body plan that characterizes a group of organisms and especially a major taxon (such as a phylum)” (Merriam-Webster, 2020b).

Founder effect: “The effect on the resulting gene pool that occurs when a new isolated population is founded by a small number of individuals possessing limited genetic variation relative to the larger population from which they have migrated" (Merriam-Webster, 2020c).

Pluripotent: “Not fixed as to developmental potentialities especially : capable of differentiating into one of many cell types” (Merriam-Webster, 2020d).

Evolution of mutability contributes to the canalization of bauplans (Box 1), which encode information about a set of evolvable genetic parameterizations of viable phenotypes in a given environment. This is an example of a meta-learning mechanism in macro-evolution.

- **Micro-evolutionary mutation within the constraints of a bauplan, allows rapid adaptation and speciation within a clade, due to well constrained search**.
  i.e., the bauplan consists of :
  (i) the set of genes that are locked down with low mutability, and
  (ii) the set of genes of high mutability that can be varied safely to produce variations within the space of viable phenotypes.e.g., the variation between breeds of a species, and the species of a genus.
- **The macro-evolutionary process of discovering and incorporating information into new parameterizations of the bauplan is very much slower, due to unconstrained search for which genes to mutate**.
  i.e., one or more locked down genes must
  (i) mutate to a high mutability state, (which may include gene duplication) and
  (ii) mutate to a new cis-regulatory state, (which may include sensitivity to different trans-regulatory factors), such that a new class of phenotype has increased fitness for some available niche.
  e.g., the emergence of lungs, dentition types, hair, feathers, paws, hooves, wings

Note, that by itself increasing mutability of previously locked down genes decreases the average fitness of offspring due to allowing non-viable mutations. For this reason radical innovations are normally associated with (i) initially less-critical body parts, and (ii) a sequence of environments and selection pressures leading to exaptation, i.e., repurposing of adaptations to new niches. When the new niches make the new adaptations critical to viable phenotypes, then there is selection pressure to reduce mutability, thereby locking down a new bauplan. e.g., the emergence in therapod dinosaurs of “flight” feathers used in incubating eggs (Norell et al., 1995; Hopp and Orsen, 2004), subsequently enabling the emergence of flight in the paraves subgroup of therapods (early birds of the Jurassic).

Implementation of mutation-of-mutability in an artificial genome would not require the 98% non-coding DNA of biological DNA. It could more tractably be implemented as one additional evolvable parameter per gene. For further details see section 3.1.

### 2.2. Morphogenesis—By Epigenetic Control of a Swarm of Cells

In the morphogenesis of multi-cellular animals, the genome plus epigenetics, control cell behavior. The phenotype of the multi-cellular organism arises from the collective behavior of cells.

#### 2.2.1. Epigenetic Cell Lines—Morphogenetic and Histological Identities

In multi-cellular organisms (with a few exceptions) every cell contains the same genetic code, but different regions of the genome are turned off, producing distinct cell types (Allshire and Madhani, 2018). These cell types respond differently to the same stimuli, and lay down different tissues in the formation of the body. Behind the behavioral cell types is a larger branching tree of epigenetic cell types, that mark the cell's line of descent from the zygote.

The mechanism behind cell lines is epigenetic regulation of gene activity, by “trans-regulatory” molecules that bind to the cis-regulatory DNA. All of these molecules, or the enzymes that make them, are coded for by regulatory genes. The interaction of genes produces gene regulatory networks (GRNs). GRNs have been used in evolutionary robotics (Doursat et al., 2013), however certain features of biological GRNs and epigenetics are important.

Firstly, there are distinct mechanisms for permanently silencing genes, such that deactivation is copied in DNA duplication and therefore inherited in all descendant cells within the organism (These epigenetic controls are mostly reset in the formation of the zygote, restoring the pluripotency of stem cells in the new embryo). The most prominent of these mechanisms is DNA methylation, leading to super coiling of the DNA into “heterochromatin” (Allshire and Madhani, 2018). This makes deactivated regions of the genome inaccessible to further interaction, and therefore enforces the non-reversibility of cell specialization (under normal *in-vivo* conditions). Non-reversibility imposes a branching tree structure on the steps of cell specialization, which produces generally predictable behavior, as opposed to the potentially chaotic behavior of non-canalized cell types.

**Figure 1:**
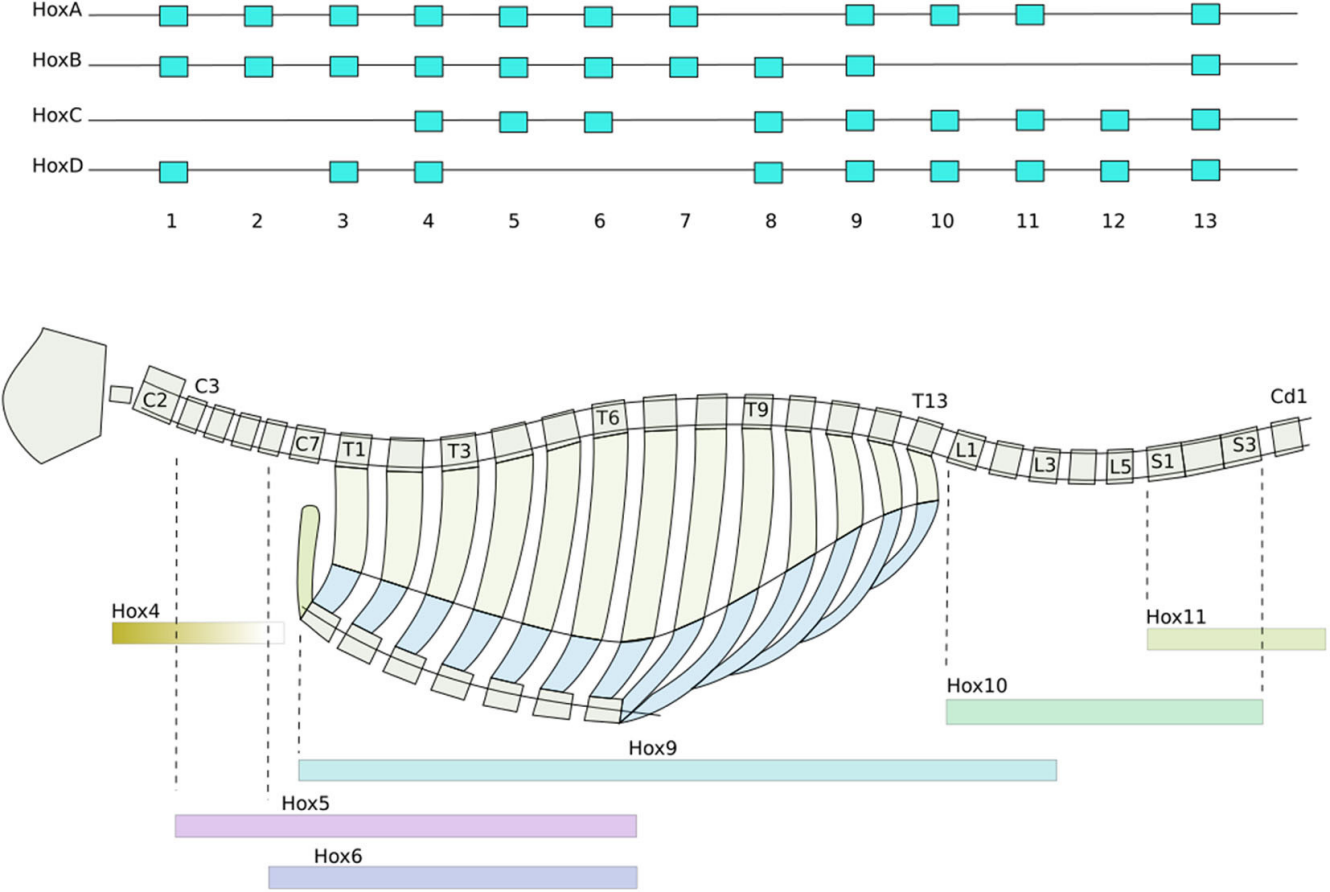
Hox gene patterning of the axial skeleton. From drawn from data in Baker et al. (2008) and Wellik (2007). **(Top)** The sequential ordering of the Hox genes preserved across all four duplicates of the cluster. Activation and deactivation of the Hox genes spreads progressively along the clusters, concurrent with the formation of the somites (Box 2). Consequently different groups of somites have different combinations of Hox genes activated. **(Bottom)** How Hox genes determine how the tissues grow, producing the characteristic types of vertebrae, collar bones, ribs, sternal manubrae, sacrum, and pelvis of the axial skeleton. Color bars indicate which hox genes are essential for each section. Each vertebral segment corresponds to a somite produced in Figure 2.

**Figure 2:**
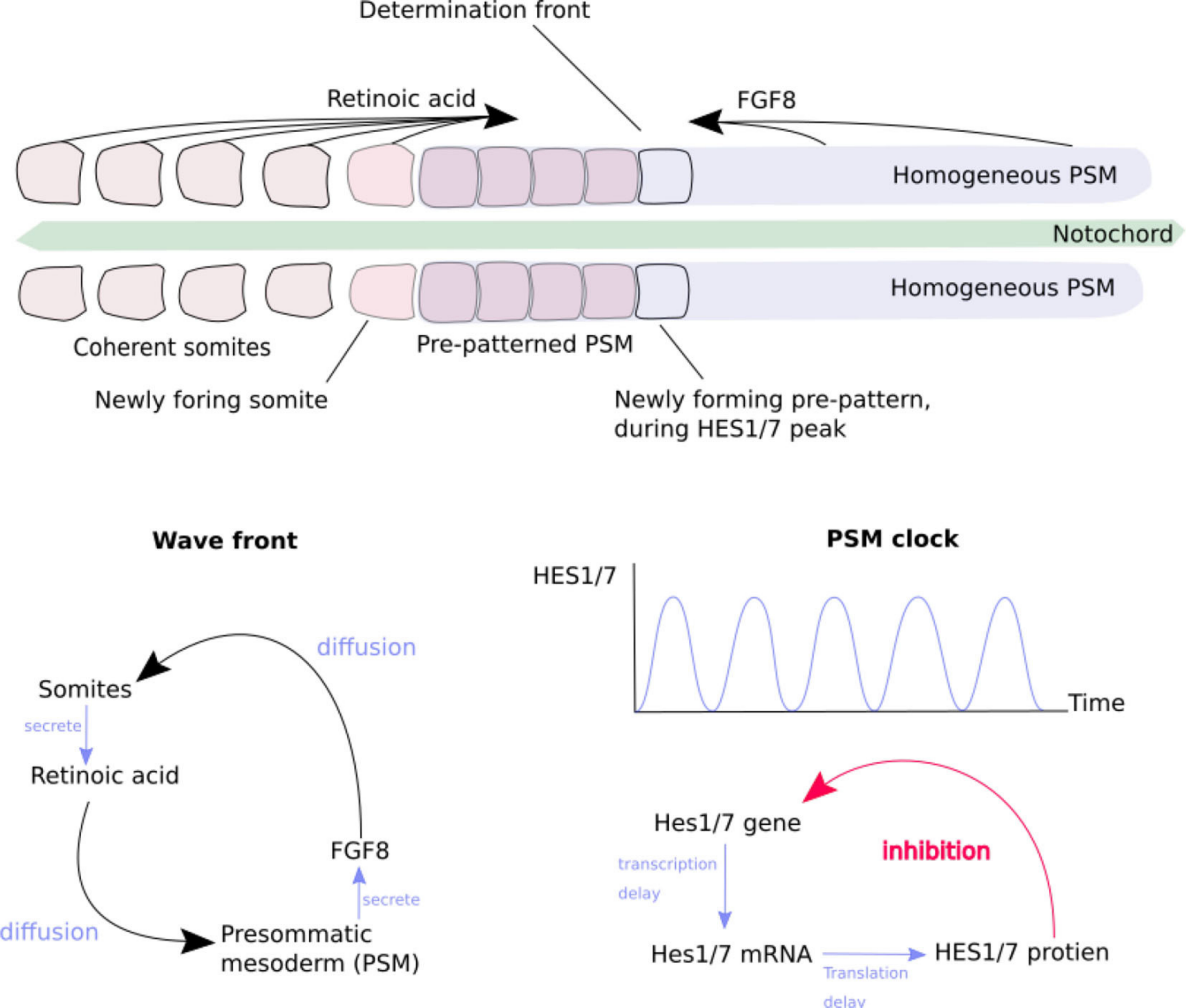
The generation of somites by the “clock-and-wavefront” mechanism of morphogens. Redrawn from Baker et al. (2008) (Figure 1). **(Top)** The progression of somite formation in the pre-somatic mesoderm (PSM) (Box 2) either side of the notocord, in early morphogenesis of a vertebrate embryo. Opposing morphogen diffusion gradients define the “determination front” which is capable of forming a new somite. **(Bottom left)** The generation of the morphogen gradient by reciprocal stimulation of PSM and somites. **(Bottom right)** The generation of the “somitogenetic clock” in the PSM, by delayed self-inhibition.

Secondly, Homeobox genes are regulatory genes responsible for epigenetic cell-type identity and body patterning. The Hox genes are a set of Homeobox genes responsible for determining the types of body segments (vertebra and associated tissues) along body axis (Mallo et al., 2010) (see Figure 1). These use a characteristic of the heterochromatin silencing mechanism. The Hox genes are arranged in sequence along a stretch of the genome. Gene silencing is initiated at one end of the Hox gene cluster, and spreads progressively along the length of cluster during formation of the body axis. As each body segment is formed (Figure 2), the spreading of inactivation is halted in that segment. Consequently different segments along the body axis have different numbers of Hox genes deactivated—so defining their epigenetic cell type as belonging to a particular region of the body. This determines the types of body segment (skull, neck, thorax, abdomen, sacrum, tail) and where the limb buds form.

Box 2 **Definitions of biological terms.**

Somatic: “Of, relating to, or affecting the body especially as distinguished from the germplasm” (Merriam-Webster, 2020e)

Mesoderm: “The middle of the three primary germ layers of an embryo that is the source of many bodily tissues and structures (such as bone, muscle, connective tissue, and dermis)” (Merriam-Webster, 2020f)

PSM pre-somatic mesoderm: “The region of mesoderm which subsequently forms the body.”

Notochord: “A longitudinal flexible rod of cells that inthe lowest chordates (such as a lancelet or a lamprey) and in the embryos of the higher vertebrates forms the supporting axis of the body” (Merriam-Webster, 2020g).

Somite: “One of the longitudinal series of segments into which the body of many animals is divided” (Merriam-Webster, 2020h).

The functions of permanent gene silencing and the spread of gene silencing could be efficiently implemented for evolutionary robotics by two variables and one parameter on genes:

- available/silenced—epigenetic variable for gene availability
- spread/stop—epigenetic variable for silencing progression
- delay/insulator—genetic parameter that determines the possibility and rate of spread of silencing.

For further details see section 3.1.

#### 2.2.2. Local Anatomical Coordinates—From Morphogen Gradients

One of the significant characteristics of animal bodies is the way their proportions can be varied, while keeping all interacting parts in their correct topology and relative positions. This implies that they are parameterized by local anatomical coordinates. The mechanism underlying this, is the control of gene activity via the secretion and diffusion of morphogen hormones through the tissues. This creates local concentration gradients that indicate the distance and direction from the tissue secreting the morphogen. Such systems of secreting cells can be self-organizing when cells of the same type have a non-linear response to the morphogen they secrete. If low concentrations of the morphogen inhibit secretion, then one cluster of secreting cells will be selected.

This mechanism enables several useful effects (Delgado and Torres, 2016; Hiscock et al., 2017). The bilateral symmetry of the body is created by breaking omni-directional symmetry, by the secretion of cranio-caudal and dorso-ventral morphogen gradients. A morphogen which at higher concentrations inhibits its own secretion can create an oscillator, which acts as a clock for the rest of the body. A morphogen which slowly activates secretion in neighboring cells produces an advancing wavefront. The combination of a clock signal and a wavefront is used to produce the basic segmentation of the vertebrae (Figure 2). The Hox gene system, section 2.2.1, is overlaid on this, producing different types of vertebrae (Figure 1).

The system of morphogens is used repeatedly in consecutive stages of fetal growth, producing a hierarchy of details patterned within tissue layers and types produced by earlier stages. This produces the basic topological arrangement and connections of body structures. For instance tendon and muscle primordia are located with respect to bones and the overlying dermis, connected to each other and to their sites of origin and insertion in the bones, under the guidance of concentration gradients and cell-type recognition on contact.

Implementation of this mechanism for evolutionary robotics requires that chemical diffusion be modeled as cell-to-cell, rather than through homogeneous space. This causes concentration gradients to conform to tissue boundaries and the body envelope. For further details see section 3.2.

#### 2.2.3. Remodeling

A critical characteristic of animal bodies is the way that kinematic chains remain minimized in mass, balanced in strength and matching geometry of all their parts (Figure 4). This holds for both genetic variation between individuals, and over the life of individual organisms as they grow, gain, and loose mass.

However, there is something more subtle than this going on. The details of geometric shape and material composition of body parts are not fixed by the genome. The shape and composition also depends on the history of forces applied. This means that movement and muscle contraction are required for morphogenesis. The genome specifies rules for how shape and composition are remodeled, through cell behavior, in response to forces.

**Figure 3:**
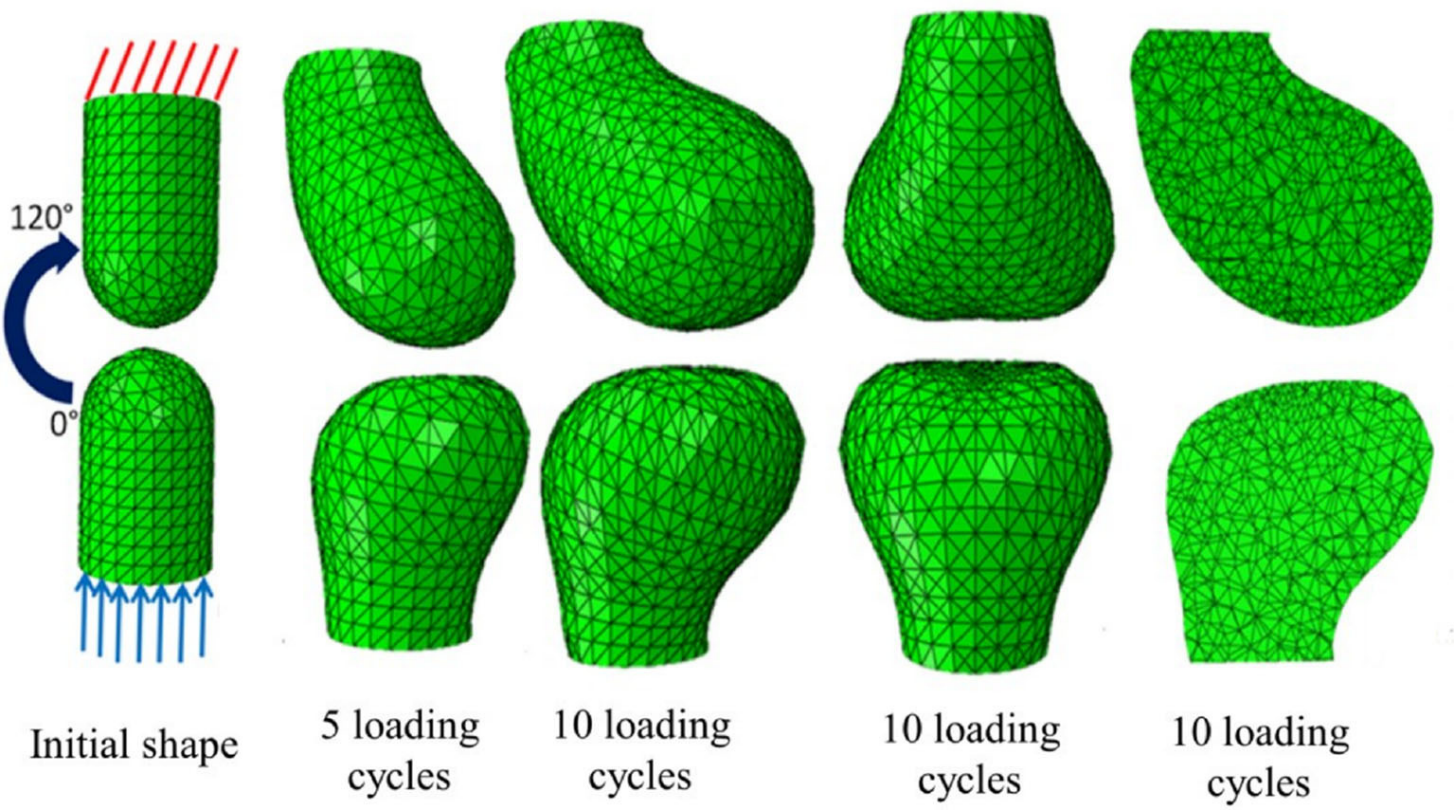
Passive mechanical shaping of bone precursors. Reproduced with permission from Giorgi (2015) *Mechanobiological predictions of fetal joint morphogenesis* (Figures 3–25). Single plane motion from 0 to 120 degrees mimicking a hinge movement; the top phalange acquired a more rounded convex profile whereas the bottom phalange acquired a flatter profile.

**Figure 4:**
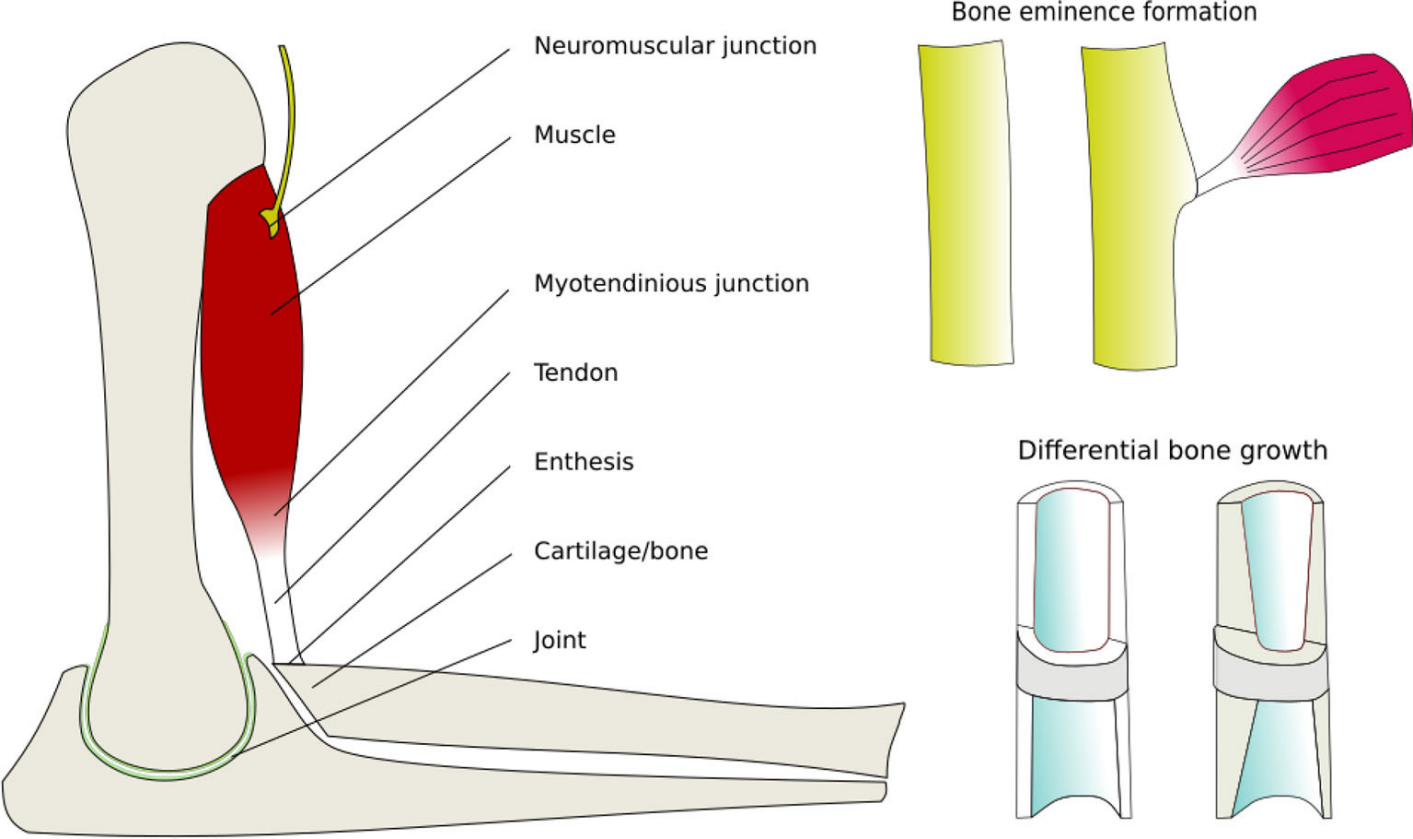
Active mechanical regulation of musculoskeletal development. Drawn from data in Felsenthal and Zelzer (2017). Components of the musculoskeletal system that are actively remodeled by cells in response to mechanical forces, thereby producing balanced strength and proportions.

Different cell types respond by secreting or resorbing their characteristic materials. *The general pattern of response is* (i) prolonged tension causes lengthening, (ii) cyclical loading causes strengthening, (iii) low peak strain causes shortening of fibrous tissue, (iv) low peak stress causes weakening. Bone acts as a master tissue, stretching other tissues to match its geometry. Bone growth is regulated by morphogen diffusion at the growth plates and articular cartilage. Bone itself is shaped by passive deformation of the bone primordia in the formation of the joints (Figure 3), and active remodeling in response to forces to form the ridges and protrusions where major muscles and tendons attach (Figure 4).

This has several important effects:

- The net result of remodeling is considerably more complex geometry and material composition than is specified in the original patterning by morphogens.
- The regulation of remodeling incorporates environmental information to ensure well matched functional body parts under genetic and environmental variation.
- It also provides a parameterization of the weight and strength of body parts, that produces smooth variation of phenotype and fitness, which is therefore evolvable.

Implementation of remodeling for evolutionary robotics would require a genome that specifies cell behavior in response to forces, and a simulation environment that models the forces generated in the tissues. For further details see 3.2.

## 3. Assembling a System

### 3.1. Genome for Cell Behavior

**Characteristics of the genome**

- The genes would need to be arranged in sequence on chromosomes, in order for “spreading of (in)activation” to be meaningful.
- Each cell would need to hold epigenetic state.
- Each major time step, the active behavior of the cell and its epigenetic state would be processed.

Each gene or cluster of genes would have several variables and parameters:

**Epigenetic variables**

- Current activation
- Available/silenced—epigenetic variable for gene availability
- Spread/stop—epigenetic variable for silencing progression.

**Genetic parameters**

- Mutability
- Delay/insulator—genetic parameter that determines the possibility and rate of spread of silencing
- Sensitivities to regulatory inputs (morphogens, stress, and strain cycles)—altering current activation
- Cell actions—secrete morphogens, move, adhere, divide, secrete/resorb material—dependent on current activation.

### 3.2. Morphogenesis Simulator/Engine

The morphogenesis engine would need to model (i) contact between cells, (ii) propagation of forces, (iii) cell-to-cell diffusion of morphogens, (iv) division of cells, (v) secretion, and resorption of different structural materials. Given the need to generate forces for remodeling, the tissues would need to include (vi) muscles/actuators and (vii) nerves to control them. While MapDevo3D (Doursat and Sánchez, 2014) already implements many of these features, it lacks the scale and speed required.

The simulator would resemble particle based multi-physics simulators such as Nvidia's “Flex” (Macklin et al., 2014). Given the need to modify the source to add the required features, an open source code would be needed as the start point. This would also allow the code to be shared with other researchers for validation of results. The open source codes DualSPHysics (Crespo et al., 2015) and Fluids-v3 (Hoetzlein, 2014) could be good start points as they support real-time simulations of millions of particles on GPUs. This is important because adequate spatial resolution is required for modeling of complex organisms/robots, and tractability is essential for evolutionary simulations.

### 3.3. The Reality Gap

All simulations need to be concerned with the ability to make useful predictions outside the simulation. While the laws of physics are not seriously in doubt for macro-scale modeling, bifurcations of behavior lead to sensitive dependency on initial conditions. This means that simulations at tractable scale and speed inevitably diverge in a short time from any single experiment. The same also applies to attempts to repeat the physical experiment—e.g., throwing a bucketful of balls.

A second issue is that simulation models are inevitably very much simpler than real materials, objects, and built machines. As above, duplicating the real machine also diverges from exact replication, such that duplicates also diverge from each other in their behavior. Consequently, the best that a simulation can achieve is to predict the distribution of behavior of the set of real objects approximating (to some chosen accuracy) the measurements that define the simulation model.

Where duplicate machines agree with each other, and with simulations, is (i) the order of magnitude of their behavior, and (ii) the topology of the bifurcations of their behavior. That is they respond in a similar way to the same control inputs, despite differing in some degree. This similarity makes it possible to control a machine despite having an imperfect model of its behavior. It also makes it possible to predict that a machine built from a simulation, would be controllable in reality with adjusted parameters.

A final concern is over-optimism about the achievable performance of components—e.g., frictionless actuators, slip free surfaces and unbreakable materials may be simple to simulate, but they cannot exist in reality. An advantage of the soft-matter simulation proposed is that it facilitates plausible constraints on material properties.

### 3.4. Design and Evolution of Bauplans

Animals have fewer genes and are therefore substantially simpler than was anticipated before the era of genomics. Most vertebrates have approximately 20,000 genes (Prachumwat and Li, 2008). The most diverse phylum, arthropods, have slightly fewer genes on average (Alfsnes et al., 2017). Of these most relate to the biochemistry of cells, rather than the morphogenesis of multi-cellular bodies.

Let us optimistically suppose that only 1,000 genes are sufficient to account for the critically necessary information for animal morphogenesis, and that an evolved robot might use as few as 100 genes. The space that needs to be searched is not *n*^100^ where *n* is the number of possible values for each gene, but *the set of all possible spaces of size* *n*^100^. That is, each of the different possible ways of parameterizing the morphogenesis of the robot in 100 or fewer genes. Given the need to compute the phenotype specified and its fitness by some relevant metric, for each sample of the genotype spaces, it will remain intractable to search a space of this size for the foreseeable future.

Given the size of the search space, it is not surprising that it took billions of years for multicellular metazoan life to evolve, after the emergence of complex eukaryotic single celled organisms. Likewise in artificial evolution, despite the relative simplicity of the genomes used, one should not expect bauplans to emerge from tabula rasa in tractable time.

Useful and interesting bauplans can be written by tracking the morphogenesis of clades that produce phenotypes of interest, e.g., chordates (vertebrates), arthropods, molluscs, and their subfamilies. This would:

1. use GRNs to create a branching tree of epigenetic cell lines, which determine sensitivities to morphogens.
2. set up local morphogen gradients to provide local anatomical coordinates.
3. locate tissue primordia, connect kinematic chains, grow, actuate, and remodel to produce a working body.

Materials and techniques for building robots based on such anatomical structures were presented in Hockings (2016). This incudes (i) an explanation of the nature of anatomical knowledge for engineers (section 1.2), and (ii) how to emulate the material and mechanical properties of anatomy with synthetic materials (section 1.3). Of particular importance are the fibrous composite nature of tissues, the elastomeric properties of the various tissue matrices, and how the topology of fibers creates mechanisms.

### 3.5. Application in Evolution of Real Robot Structures

The system described provides a very compact representation of soft robot phenotypes, comparable to swept splines but with the advantages of remodeling and conservation of topology. Hierarchical complexity specified through recursive subdivision by a branching tree of epigenetic cell types, provides a robust and intuitive way to design evolvable robot bauplans.

#### 3.5.1. Short Term

##### 3.5.1.1. MapDevo3D

It is immediately possible to use MapDevo3D to develop GRNs for morphogenetic specification of phenotypes, within its tractable scale of simulation. This would allow progress on specifying tissue types and topologies. Such models could target existing soft robot manufacturing techniques such as cast silicone pneumatics, or provide an initial approximation of mechanisms from anatomy. An important topic that could be start to be tackled is the co-development of body and nervous control during morphogenesis.

##### 3.5.1.2. GPU simulator

The lead author is currently writing an open source GPU accelerated simulator to implement the features listed in section 3.2. This is expected to substantially increase the tractable scale, allowing more complex phenotypes, as well supporting remodeling and anisotropic fibrous tissues. This should allow the morphogenesis of most musculoskeletal and dermal structures to be simulated.

For the morphogenetic mechanisms specifying the development of tetrapod vertebrate limbs including their bones, joints, muscles, tendons, and references to the primary research, see the section on “complex structures” in Hockings and Howard (2019).

##### 3.5.1.3. Hand lay-up

The immediately available way to build such structures would be hand lay-up with thermoplastic elastomer matrices (Hockings, 2016). Two critically lacking technologies are (i) somatosensory nervous system, and (ii) an actuator matching the mechanical characteristics of skeletal muscle. Despite these limitations, it would be possible to design and evolve bauplans for important robot structures such as hands and feet, where actuation and therefore proprioception (sensation of musculoskeletal pose) could be externalized via tendons to conventional actuators. Such structures would allow refinement of the technology of layered fibro-elastic mechanisms, shaping of ligamentous joints, and the selection of materials for constructing them.

#### 3.5.2. Medium Term

##### 3.5.2.1. Automatic lay-up

Hand layup is laborious and imprecise. Automating the full pipeline from simulation to CNC lay-up is an important priority. This requires both physical hardware (fiber and matrix laying tools) and the software pipeline to convert a simulated phenotype into commands for a manufacturing robot.

The lead author is currently developing a fiber laying head that can impregnate a fiber tow with different matrices along its length. This is required to anchor a ligament or tendon into a rigid structure. This could be used either to produce flat layers on a 3D printer, to be wrapped by hand onto the robot being built, or mounted on a robot arm for fully automatic building of physical copies of simulated phenotypes. It is intended to develop “slicing” for fiber laying as a function of the simulator being developed.

##### 3.5.2.2. Sensors and actuators

The most direct route to adaquate somatosensory and actuation technology would be bulk-MEMS systems with a feature scale 1-10 microns, on a flexible substrate. Lift-off processing of photo-patternable polyimide and silicones would allow such devices to be made in university photolithography labs. Once proven these techniques could be scaled up by roll-to-roll contact lithography.

A viable somatosensory system might use thermal and mechanical sensors 100 microns external size, connected via stretchable (sinusoidal) fibers 20 microns wide, converging to the pixels of a video chip. This would allow processing somatosensory data with existing computer vision techniques.

The most immediate way to match the cycle speed, effective strain and work-per-stroke-per-unit-mass of skeletal muscle would likely be a reduced feature size version of the Dual Excitation Multiphase Electrostatic Drive (Niino et al., 1995; Yamamoto et al., 1998). If made with 5 micron feature scale and layer thickness, this could be macroscopically fibrous. With each fiber encased in elastomer, it would be packable like muscle fiber.

#### 3.5.3. Longterm

##### 3.5.3.1. New materials

An important addition will be new materials that may facilitate manufacture. In particular design of brush-polymers to provide intrinsically conductive thermoplastic elastomers, controlled adhesion/nonadhesion, stable cross-linking, and self-healing. These would facilitate components such as fiber-drawn rolled dielectric elastomer actuators for fully soft muscles and sensors.

##### 3.5.3.2. Automated design

While evolution uses only the information implied in the genome, design can access other information to constrain the search, including knowledge of physics, materials, and multiple levels of abstraction. This is why engineering design is so much faster and capable of leaps across evolutionary barriers. The development of artificial intelligence capable of minimally supervised learning, development of technologies, and the rapid generation of useful high performing designs is the most important long term goal. In the context of techniques in this paper, given a problem description such a system would write novel evolvable bauplans.

## 4. Conclusion

Especially when looking at robotic forms, evolutionary robotics has in the past based its encodings on biology. However, overall, the depth of modern biological understanding has not been translated into the evolutionary robotics literature that it inspires.

Here we have shown some currently unused mechanisms from evolutionary biology, and provided guidelines for their transfer to evolutionary robotics. We hope that this inspires evolutionary robotics researchers to immerse themselves more fully into the biological literature, which could open up new avenues of research and heightened capabilities for evolutionary robotics in general.

## Author's Note

This article is intended for expert discussion, and requires prior knowledge of terms and concepts in biology and information theory.

## Author Contributions

DH devised the project. NH wrote the manuscript in consultation with DH. Both authors contributed to the article and approved the submitted version.

## Conflict of Interest

The authors declare that the research was conducted in the absence of any commercial or financial relationships that could be construed as a potential conflict of interest.
